# Tapasin gene polymorphism in systemic onset juvenile rheumatoid arthritis: a family-based case–control study

**DOI:** 10.1186/ar1480

**Published:** 2005-01-11

**Authors:** Hulya Bukulmez, Mark Fife, Monica Tsoras, Susan D Thompson, Natalie A Twine, Patricia Woo, Jane M Olson, Robert C Elston, David N Glass, Robert A Colbert

**Affiliations:** 1 William S. Rowe Division of Pediatric Rheumatology, Cincinnati Children's Hospital Medical Center, University of Cincinnati, Cincinnati, Ohio, USA; 2 Department of Epidemiology and Biostatistics, Case Western Reserve University, Cleveland, Ohio, USA; 3 Department of Genetics, Case Western Reserve University, Cleveland, Ohio, USA; 4 Pediatric Rheumatology, Pediatrics, Metro Health Medical Center, Case Western Reserve University, Cleveland, Ohio, USA; 5 The Center for Pediatric and Adolescent Rheumatology, University College London, London, UK

**Keywords:** association, linkage, juvenile rheumatoid arthritis, tapasin, *TPSN*, transmission disequilibrium testing

## Abstract

Juvenile rheumatoid arthritis (JRA) comprises a group of chronic systemic inflammatory disorders that primarily affect joints and can cause long-term disability. JRA is likely to be a complex genetic trait, or a series of such traits, with both genetic and environmental factors contributing to the risk for developing the disease and to its progression. The HLA region on the short arm of chromosome 6 has been intensively evaluated for genetic contributors to JRA, and multiple associations, and more recently linkage, has been detected. Other genes involved in innate and acquired immunity also map to near the HLA cluster on 6p, and it is possible that variation within these genes also confers risk for developing JRA. We examined the *TPSN *gene, which encodes tapasin, an endoplasmic reticulum chaperone that is involved in antigen processing, to elucidate its involvement, if any, in JRA. We employed both a case–control approach and the transmission disequilibrium test, and found linkage and association between the *TPSN *allele (Arg260) and the systemic onset subtype of JRA. Two independent JRA cohorts were used, one recruited from the Rheumatology Clinic at Cincinnati Children's Hospital Medical Center (82 simplex families) and one collected by the British Paediatric Rheumatology Group in London, England (74 simplex families). The transmission disequilibrium test for these cohorts combined was statistically significant (χ^2 ^= 4.2, one degree of freedom; *P *= 0.04). Linkage disequilibrium testing between the HLA alleles that are known to be associated with systemic onset JRA did not reveal linkage disequilibrium with the Arg260 allele, either in the Cincinnati systemic onset JRA cohort or in 113 Caucasian healthy individuals. These results suggest that there is a weak association between systemic onset JRA and the *TPSN *polymorphism, possibly due to linkage disequilibrium with an as yet unknown susceptibility allele in the centromeric part of chromosome 6.

## Introduction

Juvenile rheumatoid arthritis (JRA) is the most common chronic arthritic condition of childhood, encompassing pauciarticular, polyarticular, and systemic-onset disease subtypes. JRA is typically considered autoimmune in etiology, with characteristic T-cell abnormalities and chronic synovitis. The extent of synovitis may range from minimal to severe, and vary in terms of number of joints involved, with systemic onset disease typically associated with the greatest morbidity. JRA is probably a collection of diseases with complex overlapping etiologies, with each subtype influenced by multiple genetic susceptibility loci and mediated by environmental effects [1]. The MHC on the short arm of chromosome 6 has been intensively analyzed, and associations with both HLA and non-HLA genes have been reported. Genetic associations with MHC alleles have been documented primarily within the HLA class II region, but also with certain class I alleles. These associations are largely JRA subtype and age specific [2], and are strongest for pauciarticular and polyarticular disease [1]. For systemic onset JRA (SoJRA), associations with *HLA-B8*, *HLA-Bw35 *[3,4] and *HLA-DR4 *[3,5] have been observed, whereas HLA-DPB1*0401 was reported to have a protective effect in one Caucasian population [6]. Associations with HLA-DRB1*0401 and HLA-DRB1*0405 have been reported in a Japanese population [7]. Most of these associations have not been replicated.

In the present study we targeted the tapasin gene (*TPSN*), which is in the class II region of the MHC, 180 kilobases centromeric of HLA-DP. The tapasin protein is necessary for the proper assembly and peptide-presenting function of HLA class I molecules [8]. The *TPSN *gene has a polymorphism in exon 4 that results in a nonconservative amino acid substitution of Arg/Thr at amino acid 260 (ref SNP ID: rs2071888) [9,10]. Three intronic polymorphisms of *TPSN *have also been described, none of which appear to be in linkage disequilibrium (LD) with HLA class I alleles or the extended *HLA-A1*, *HLA-B8*, *HLA-DR3 *haplotype, in at least one healthy Caucasian population [11]. Furthermore, using a large UK Caucasian sample, Ahmad and coworkers [12] recently reported that *TPSN *polymorphisms are not in LD with more telomeric MHC haplotypes. In the present study we report an association between the exon 4 *TPSN *polymorphism and susceptibility to SoJRA, involving the *TPSN *allele Arg260 (01 allele).

## Methods

The study cohort included 88 SoJRA affected families recruited in Cincinnati (US cohort) and 74 simplex (with one affected offspring) SoJRA families identified by the British Paediatric Rheumatology Study Group (UK cohort). Unaffected siblings were available for analysis in the US but not in the UK cohort. An additional 113 healthy unrelated control individuals, primarily Caucasians from the Midwest and New England, resembled the SoJRA-affected families in terms ethnicity and served as the control population. Ethics approvals were obtained from the participating institutions, and informed consent was obtained from parents and/or children.

All affected children met American College of Rheumatology criteria for a diagnosis of JRA; they were subgrouped as pauciarticular, polyarticular, or SoJRA. Genomic DNA was purified from peripheral blood cells by standard techniques and analyzed for *TPSN *alleles (Arg260/01 and Thr260/02) by polymerase chain reaction and restriction site enzyme digestion. Briefly, a 298-base-pair fragment of exon 4 of the *TPSN *gene containing the polymorphism was amplified and then digested with *Bfa*I, which recognizes the 01 allele, and *Sfc*I, which recognizes the 02 allele. The primers used were Tsn 479 forward (5'-CCC ACC CTC TAC CCC TGG A-3') and Tsn 641 reverse (5'-CAG CAC CTG GGT AAG GGA CA-3'). HLA types were determined for a subgroup of the participants using DNA-based low-resolution methodology (Geno-Vision Inc., Exton, PA, USA), and serologically using standard typing sera and microcytotoxicity assays.

Preliminary association analysis was conducted by χ^2 ^testing on contingency tables comparing the three genotypic frequencies between cases and control individuals to yield a χ^2 ^with two degrees of freedom. Family-based association analysis was performed using the transmission disequilibrium test (TDT). The TDT [13,14] is a family-based association test that compares within a cohort the number of times a particular parental allele is transmitted to an affected offspring versus the number of times it is not transmitted. To allow inclusion of families with missing data for a single parent, the Transmit program [15], which uses population allele frequencies to weight the possible parental genotypes, was used for the TDT analysis. In the Transmit program, genotypes of unaffected siblings (or siblings whose disease status is unknown) are used to infer parental genotypes, thus increasing the power to detect association. We applied this test first to the combined US and the UK data and then to each set separately. The significance level of the combined results was also calculated using Fisher's method of combining *P *values for two independent analyses that test the same hypothesis. LD between *TPSN *and the HLA region (limited to the Cincinnati cohort) was evaluated using the EH program [16,17]. The Geno-Pdt test in the PdT 5.1 program  was also used as a test for association and linkage in the US SoJRA-affected families [18-20].

## Results

The distribution of tapasin genotypes among SoJRA-affected children was compared with that in their healthy siblings, as well as with that in unrelated healthy control individuals using a two degrees of freedom χ^2 ^test in the US cohort (Table 1). The differences did not reach statistical significance. The allelic frequencies of tapasin in the independent cohorts from the USA (Table 1) were in Hardy–Weinberg equilibrium (healthy individuals from the USA: χ^2 ^= 0.3049, *P *= 0.58; SoJRA-affected individuals from the USA: χ^2 ^= 2.004, *P *= 0.156). In the UK data only SoJRA-affected individuals were available for Hardy–Weinberg equilibrium testing, and the result was borderline (χ^2 ^= 5.26, *P *= 0.02).

We then tested for evidence of linkage of *TPSN *to JRA by applying the family-based TDT only to the cohorts of affected individuals for whom parental and sibling information was available. These families included 82 US SoJRA familes (389 individuals, including family members) and 74 UK SoJRA families. The TDT test, as implemented in the Transmit program, detected the preferential transmission of the *TPSN *01 allele in the UK and US SoJRA families (*n *= 156; χ^2 ^= 4.2, 1 degree of freedom [df]; *P *= 0.04; Table 2). When the US SoJRA cohort was analyzed alone, this preferential transmission for the *TPSN *allele 01 was even more significant (χ^2 ^= 6.0, 1 df; *P *= 0.01; Table 2).

However, SoJRA families from the UK alone as a subgroup failed to show significant preference for *TPSN *allele 01 transmission (χ^2 ^= 0.075, 1 df; *P *= 0.78; Table 2). When the SoJRA UK cohort was analyzed, it was recognized that information for one of the parents was missing in 22 families (29.7% of the total). There was no information from unaffected siblings of the probands for these UK families, which is necessary for the Transmit program to narrow down the range of the possible parental genotypes. When one parent is missing, the Transmit program assigns and weights possible haplotypes to the missing parent using the information from the known parent and the siblings of the proband, and averages the probability of all transmissions to the proband. Because there were no data regarding the genotypes of unaffected siblings for the UK families, the Transmit program was unable to infer parental genotypes and thus had less power to detect the preferential transmission of the 260Arg allele. In the US SoJRA-affected cohort parental information was missing for 32 of the 82 families (39%), but for 12 of these (14.6%) there was information regarding unaffected siblings. Although 24.4% of the cohort was unavailable for TDT calculation, 62 families (75.6%) were available.

We combined the two *P *values using Fisher's method [21] to obtain a χ^2 ^with four degrees of freedom and found the combination of the two analyses to be significant (χ^2 ^= 9.7, 4 df; *P *= 0.05). Overall, when the two cohorts were analyzed together the tapasin 260Arg allele was transmitted more often than the 260Thr allele, suggesting association and linkage between the *TPSN *polymorphism and SoJRA. In order to include information from the unaffected siblings for association testing, we also applied an alternative association test to the US SoJRA population – the pedigree disequilibrium test (PDT) [18,19]. A new version of PDT, the genotype-based association test for pedigrees (Genotype-PDT), was applied to the data. Genotype-PDT [20] tests for linkage and underlying patterns of association at the genotypic level. It is more conservative and has lesser type 1 error when compared with the TDT test implemented in the Transmit program. Genotype-PDT also uses information from affected individuals, unaffected siblings, and their nuclear families. Therefore, we were only able to apply this test to the US SoJRA cohort. The genotype-PDT test revealed association and linkage of the tapasin 260Arg allele with SoJRA at the genotypic level (χ^2 ^= 6.727, 1 df; *P *= 0.034) in the US SoJRA cohort.

Furthermore, we wished to control for possible transmission distortion of tapasin 260Arg allele in SoJRA-affected families. This allele could also be preferentially transmitted to the unaffected siblings of the SoJRA-affected individuals from their parents, and our statistical significance could be falsely inflated because of allele-specific segregation bias (altered transmission of an allele independent of its role in disease). We therefore applied the TDT test to the unaffected siblings from the US SoJRA cohort. In contrast to the affected siblings, there was no significant preference toward 260Arg allele transmission to healthy siblings, suggesting no segregation bias (χ^2 ^= 1.043, 1 df; *P *= 0.3; Table 2). These data provide evidence of a genotypic association and linkage between the *TPSN *260Arg allele and susceptibility to SoJRA.

## Discussion

Although JRA is the most common rheumatologic disease in childhood, the SoJRA subtype comprises less then 20% of cases and is a rare disease. In the past, because of the small sample sizes, studies conducted by single centers failed to establish strong genetic associations. The present study was therefore done with collaboration between two different centers. These two centers recruited mainly Caucasian families with one SoJRA-affected offspring. An association of the *TPSN *allele 260Arg with SoJRA was detected when both cohorts were analyzed and in the US cohort by itself. In the UK cohort the statistical analysis did not reveal a significant association. This discrepancy may be due to the limited sample size for the UK data, the different ethnic backgrounds of the two cohorts, and/or gene–environment interactions.

In general, it is suggested that studies using independent controls are more powerful than those using related (family-based) controls, but they may be biased if cases and controls have different ethnic backgrounds because of population stratification. Family-based control studies are less powerful because of overmatching, but they are robust to population stratification. In the present study we used family-based control association tests, which allowed us to analyze SoJRA family cohorts recruited by two different centers (US and UK).

Statistical programs designed to test genetic linkage based on TDTs (i.e. linkage in the presence of association) calculate the transmission of alleles from heterozygous parents to affected individuals. In the absence of one parent, the family becomes uninformative regarding single nucleotide polymorphisms and cannot be included in the analysis. This decreases the sample size, thus reducing the power to detect association or genetic linkage in rare diseases. Recently, programs such as Transmit and PDT have become available that are designed to calculate the possible genotypes of the missing parent from unaffected children or other family members such as grandparents. However, in cohorts consisting of simplex families (mother, father and the affected child), which do not have unaffected siblings or grandparents, and when there are families with missing parents, these programs are unable to achieve as much power to detect genetic association. The TDT test in the Transmit program was unable to infer the missing parental information (22 families, 29% of the data) from the UK cohort, which decreased the sample size to 52 families (71% of the cohort). In contrast, in the US SoJRA cohort the presence of unaffected siblings made 62 families (75.6% of the cohort) available for testing and increased the power to detect linkage in the presence of association.

In order to detect whether the group of children with earlier age at onset of SoJRA (<6 years at onset) is in association with *TPSN *allele 260Arg, both cohorts were dichotomized by age at disease onset and analyzed using the Transmit program. *TPSN *allele 260Arg was still preferentially transmitted in both of the age onset groups but there was no statistical significance at the 5% level. When the age at onset groups <6 years and ≥6 years were pooled together from US and UK cohorts and analyzed, there was still no statistically significant association with *TPSN *allele 260Arg.

The other possible reason for the lack of significant linkage in the UK cohort when analyzed alone might be ethnic difference, with a different polymorphism associated with SoJRA and a different disease frequency. Although both cohorts consisted of Caucasians, there might still have been ethnic differences between them. Therefore, it could be that the association of *TPSN *allele 260Arg with SoJRA in the US population is due to LD, with different SoJRA susceptibility alleles located on chromosome 6 being due to differing recombination processes between the US and UK Caucasian populations.

Because weak associations between SoJRA and *HLA-DR *alleles [5-7] have previously been noted, we compared the available class II allele frequencies, including *HLA-DR*, *HLA-DP *and *HLA-DQ*, in SoJRA patients from the US cohort (*n *= 69) with healthy control individuals (*n *= 66). No statistically significant differences were found (data not shown). However, it is worth noting that we detected a small trend toward a lower HLA-DPB*0401 frequency in SoJRA patients (28%) as compared with healthy control individuals (35%), which is consistent with a previous report [6] that suggested a possible protective role for this particular HLA allele in SoJRA. Furthermore, we applied a test for LD (using the EH program) to assess LD between *TPSN *alleles and the HLA alleles in SoJRA patients. We included 34 SoJRA-affected children from the US cohort and 38 healthy individuals for whom HLA typing data were available. There was no evidence for LD between the *TPSN *and any of the HLA alleles with the SoJRA-affected individuals or the healthy individuals. Because HLA typing was not available for all patients in the US and UK SoJRA patient groups, these calculations were done using very small sample sizes, and so the possibility of LD between the *TPSN *and HLA loci in these groups cannot be completely eliminated.

The landmark cytokines that contribute directly to the clinical features or autoimmune process of SoJRA, namely IL-6, tumor necrosis factor (TNF)-α, and IL-1, are also known to be important regulators of apoptosis. SoJRA's characteristic clinical and laboratory features, such as fever, skin rash, hypergammaglobulinemia, hypoalbuminemia, elevated erythrocyte sedimentation rate, and fibrinogen levels, may all be explained by cytokine-activated inflammatory and/or immune responses. Elevated blood level of IL-6 in SoJRA is known to correlate with fever episodes [22,23]. Some of these cytokines were evaluated for their associations with SoJRA. The non-MHC cytokine gene polymorphisms that have been associated with SoJRA are the IL-6 5' flanking polymorphism [24], the TNF-α 5' flanking polymorphism [25], and the macrophage migration inhibition factor polymorphism [26]. Recent cytotoxicity studies also implicate natural killer cell dysfunction in this process [27].

Functional differences between the tapasin proteins encoded by the two alleles (Arg versus Thr at 260) have not, to our knowledge, been described. Given what is known about the function of tapasin, it is conceivable that polymorphisms might affect the quality or quantity of peptides presented by class I molecules, thereby influencing the immune response. It is also worth noting that the *TPSN *gene is separated from *Daxx*, an effector of Fas ligand and transforming growth factor-β mediated apoptosis [28,29] by only a single gene (*BING2*). Apoptosis plays a key role in regulating the immune response in part by balancing excess cellular proliferation, and several of the key cytokines that have been implicated in the pathogenesis of SoJRA, such as TNF-β, IL-6, and IL-1, are known to influence apoptotic pathways. Thus, it is perhaps more tempting to speculate that the *TPSN *01 polymorphism (TPSN 260Arg) associated with SoJRA might be in LD with another susceptibility allele in a gene such as *Daxx *(or other genes in the region that play roles in apoptosis). Furthermore, the *TPSN *260Arg allele might be part of a haplotype in the HLA region that contributes to susceptibility to SoJRA. It will be important to examine additional SoJRA populations to determine whether TPSN is associated with disease. If so then further genetic studies of this region, including LD testing and exploration of candidate gene alleles in the region, may be of considerable interest.

## Conclusion

In conclusion, our studies support the existence of a weak association, possibly due to a linked gene in the region, between the *TPSN *01 allele and susceptibility to SoJRA.

## Abbreviations

df = degrees of freedom; IL = interleukin; JRA = juvenile rheumatoid arthritis; LD = linkage disequilibrium; MHC = major histocompatibility complex; PDT = pedigree disequilibrium test; SoJRA = systemic onset juvenile rheumatoid arthritis; TDT = transmission disequilibrium test; TNF = tumor necrosis factor.

## Competing interests

The author(s) declare that they have no competing interests.

## Authors' contributions

HB carried out the molecular genetic study in US, genotyped the US cohort, did the statistical analysis of both US and UK cohorts, and drafted the manuscript. MF carried out the molecular study in UK and participated in drafting the manuscript. MT confirmed the genotypes of US SoJRA patients. SDT participated in the coordination of the study and drafting the manuscript. NAT genotyped the UK SoJRA cohort. PW coordinated the UK study and participated in drafting the manuscript for the UK cohort.

## Figures and Tables

**Table 1:** Tapasin genotypic distributions in systemic onset juvenile rheumatoid arthritis families

Tapasin genotypes	US healthy control individuals	US healthy siblings of SoJRA	US SoJRA	UK SoJRA	UK and US SoJRA
0101	35 (31.0%)	26 (28.0%)	49 (36.6%)	23 (30.0%)	72 (34.4%)
0102	56 (49.5%)	50 (53.7%)	63 (47.0%)	45 (60.0%)	108 (51.6%)
0202	22 (19.5%)	17 (18.3%)	22 (16.4%)	7 (9.2%)	29 (14.0%)
01 allele frequency	0.56	0.55	0.6	0.6	0.6
02 allele frequency	0.44	0.45	0.4	0.4	0.4
Total	113	93	134	76	209

Tapasin 01 allele is Arg260, and 02 is Thr260. SoJRA, systemic onset juvenile rheumatoid arthritis.

**Table 2:** Transmission disequilibrium test analysis of the US and UK systemic onset juvenile rheumatoid arthritis families using Transmit

		Arg260 transmission		Thr260 transmission			
				
SoJRA families	*n*	Observed	Expected	Observed	Expected	χ^2 ^(1 df)	*P*
US only	82	99	(88.0)	65	(75.9)	6.0*	0.01*
US unaffected siblings	79	82	(77.5)	76	(80.5)	1.04	0.3
UK only	74	91	(89.0)	57	(58.0)	0.08	0.78
UK and US together	156	190	(177.8)	122	(134.2)	4.2*	0.04*

*Significant findings. *n*, number of informative families for transmission; UK, British Paediatric Rheumatology Study Group cohort; US, Cincinnati cohort.
